# Mutations in the C-terminus of HBoV NS1 affect the function of NP1

**DOI:** 10.1038/s41598-017-06513-4

**Published:** 2017-08-07

**Authors:** Junmei Zhang, Yan Bai, Bing Zhu, Sujuan Hao, Zhen Chen, Hanzhong Wang, Wuxiang Guan

**Affiliations:** 1 Center for Emerging Infectious Diseases, Wuhan Institute of Virology, Chinese Academy of Sciences, Wuhan, Hubei 430071 China; 2 University of Chinese Academy of Sciences, Beijing, 100049 China; 3 Pediatric department of Union Hospital, Tongji Medical College, Huazhong University of Science and Technology, Wuhan, China; 4 Center Laboratory, Guangzhou Women and Children’s Medical Center, Guangzhou, 510120 P. R. China

## Abstract

Human bocavirus 1 (HBoV1) is an autonomous parvovirus in the *Bocaparvovirus* genus. The multifunctional nuclear protein NP1 is involved in viral replication. In the present study, we found that the mutations in the C-terminus of NS1 affected NP1 function in viral replication. Knocking out NP1 expression in the recombinant infectious clone, on which the C-terminus of NS1 was mutated based on the clinical samples from nasopharyngeal aspirates, resulted in different degrees of decreased replication. The result suggested that NP1 facilitated the replication of viral genome but was not necessary, which is different from the minute virus of canines, where NP1 is essential for viral replication. Further studies showed that clinical mutations in the NP1 region did not affect viral genome replication, and UP1 promoted viral DNA replication. Our results suggested that the C-terminus of NS1 is important for viral replication and may be a target for regulating the replication of the viral genome.

## Introduction

Human bocavirus 1 (HBoV1) was first identified in pooled human respiratory tract samples in 2005^1^. HBoV, bovine parvovirus (BPV) and minute virus of canines (MVC) belong to the *Bocaparvovirus* genus in the *Parvoviridae* family^1, 2^. In addition to human parvovirus B19 (B19V), HBoV1 is the second member of the *Parvoviridae* family to be potentially associated with human diseases. The prevalence of HBoV1 infection is 1.5–11.3%, and most detections occur in young children with upper or lower respiratory tract diseases^3–6^. In most cases, HBoV1 was found to be co-infected with other viruses^7^.

HBoV1 contains a single-stranded DNA genome of 5.5 kb with hairpins at both ends, which are essential for viral DNA replication. All mRNA transcripts are alternatively processed from the mRNA precursor transcribed from the P5 promoter on the left of the genomic DNA^8–10^. The left half of the genome encodes non-structural proteins NS1, NS2, NS3, NS4 and NS1-70^9, 11^. The right half of the genome encodes structural proteins VP1, VP2 and VP3^9, 10, 12^. The N-terminus of the VP1 unique region includes a phospholipase A2 (PLA2) domain, which is involved in the parvovirus infectivity^13, 14^. The open reading frame in the middle of the viral genome encodes a non-structural protein NP1^9–11, 15^.

The replication of parvovirus adeno-associated virus type 2 is proposed as a rolling-hairpin replication model^16, 17^, while parvovirus B19V adopts a hairpin-independent replication model^18^. The non-structural protein *rep* or NS1 and the hairpin structures are essential in both replication models. A unique feature of HBoV1 is the expression of the non-structural protein of NP1, which has been reported to be required for efficient viral DNA replication^9, 19^, reading through of the proximal polyadenylation site^9, 20^, regulating RNA splicing^12, 21^ and the production of VP mRNAs^9, 12, 21^.

The HBoV1 NS1 protein is a multifunctional protein that is essential for viral replication^9^. The N-terminal domain of NS1 harbors the recognition site of the viral replication origin and the endonuclease active site^22^. The ATPase and helicase domains are located in the middle of NS1^17, 23^. The C-terminal is a transactivation domain^22–25^. Non-structural proteins NS2, NS3, and NS4 are dispensable for viral replication in HEK293 cells, although these proteins contain functional domains of NS1^11^. However, NS2 is essential for HBoV1 DNA replication in primary human airway epithelium cultured at an air-liquid interface (HAE-ALI) cells^11^. NS1-70 contains the origin DNA-binding/endonuclease and helicase domains of NS1, but not the C-terminus, and the function of NS1-70 is unknown.

The left end hairpin (LEH) of HBoV1 includes 140 nucleotides (nt) and forms a rabbit’s ear structure with mismatched nucleotides. The right end hairpin (REH) forms a perfect palindrome with 200 nucleotides^9^. It has been reported that the LEH is not required for viral DNA replication, while the REH plays an important role in the DNA replication of HBoV1^26^. The replication origin of parvovirus contains Rep78/68 or NS1 binding elements (RBEs or NSBEs, respectively), which are always composed of tetranucleotide repeats and are recognized by the origin-binding domain (OBD) of Rep78/68 or NS1^27–29^. A nicking site is also located in the replication origin of either hairpin, which is normally 7 to 17 nucleotides ahead of the RBE or NSBE and is nicked by the endonuclease activity of Rep78/68 or NS1^26^.

In the present study, we found that knocking out NP1 expression by a point mutation on the recombinant infectious clone of HBoV1 (pHBoV1-WH), based on a Wuhan isolate sequence, did not affect the viral genome replication efficiency, which contradicts the previous report that NP1 is essential for viral genome replication^9^. Sequence analysis showed that there were two point mutations in the C-terminus of NS1 ORF between the Wuhan isolate and the reported Salvador isolate. NS1 and NP1 ORFs were amplified and sequenced from the clinical nasopharyngeal aspirates to further check the effect of mutations in the NS1 region on NP1 function. Several mutations were found in the C-terminus of NS1. Knocking out NP1 ORF based on the NS1 mutated recombinant HBoV1 clone resulted in the differential reduction of replication efficiency, which indicated that the C-terminus played a role in viral replication. Moreover, NP1 facilitated the replication of the viral genome and progeny virus production but was not necessary for viral DNA replication. Further study showed that clinical mutations in the NP1 region did not affect viral genome replication; however, UP1 promoted viral DNA replication. Finally, we characterized the *cis* elements required for viral genome replication. Our results suggested that the C-terminus of NS1 might be a target for regulating the replication of the viral genome.

## Results

### NP1 was not necessary for HBoV1 genome replication

**Figure 1:**
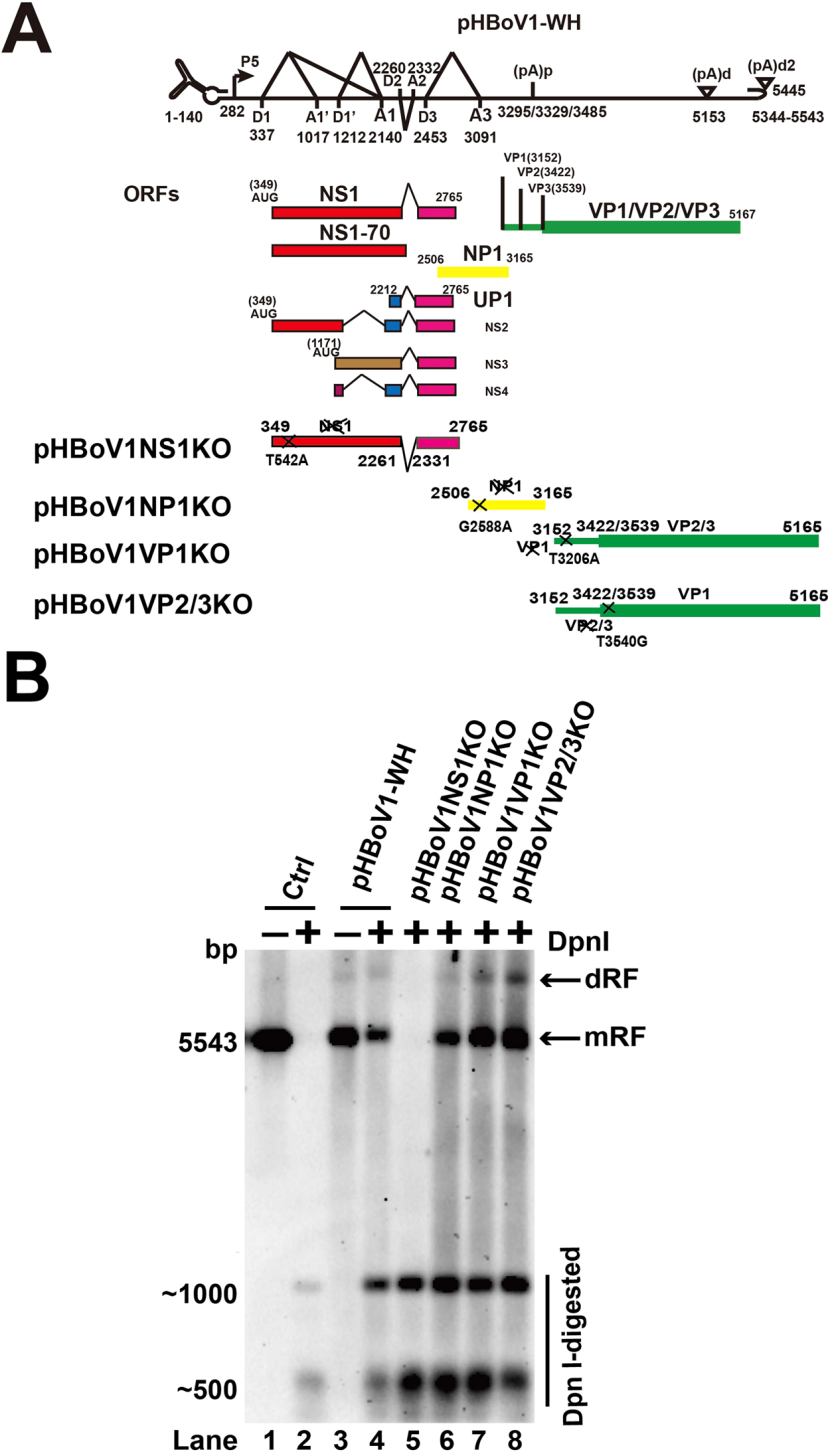
NS1 was required for HBoV1 genome replication. (**A**) Transcription map of HBoV. The P5 promoter, splicing donor sites (**D**), splicing acceptor sites (**A**), proximal polyadenylation site (pA)p and distal polyadenylation site (pA)d are depicted. The numbers denote the nucleotide positions in HBoV genome. Single nucleotide mutation to disrupt the open reading frame of non-structural (NS1, NP1) and structural protein (VP1, VP2/3) is indicated. (**B**) Southern blot analysis. HEK293T cells were transfected with plasmids as indicated and low molecular weight DNA was isolated post-transfection 48 hrs. Dpn I digested DNA fragments were separated by 1% agarose gel, followed by transferring to Hybond-N^+^ membrane. The signal was detected by hybridization with an HBoV1 genome DNA probe spanning from nt 1 to nt 5543. Lane 1 and lane 2 show the Dpn I undigested or digested bands of HBoV DNA as a control and a size marker. mRF, mono replicative forms; dRF, double stranded replicative forms.

NS1, NP1 and inverted terminal repeats (ITRs) of *Bocaparvovirus* are essential for viral genome replication^9, 19, 26^. The recombinant infectious clone (pHBoV1-WH), which can replicate in HEK293T cells and produce a recombinant infectious virus, was constructed based on the sequences from a clinical feces sample isolated from Wuhan^30^. The knockout plasmids (pHBoV1NS1KO, pHBoV1NP1KO, pHBoV1VP1KO and pHBoV1VP2/3KO) were made by single nucleotide mutation (Fig. 1A). These knockout plasmids were transfected into the HEK293T cells to identify the viral proteins necessary for the replication of HBoV1, NS1, NP1, VP1 and VP2/3. Low molecular weight DNA (Hirt DNA) was harvested and subjected to Dpn I digestion. Dpn I digests DNA that comes from bacteria. However, the replicated DNA that does not undergo methylation in the cells is resistant to Dpn I digestion. The Dpn I resistant band was not detected when pHBoV1NS1KO was transfected (Fig. 1B, lane 5), while Dpn I resistant bands were detected when pHBoV1VP1KO and pHBoV1VP2/3KO were transfected (Fig. 1B, lanes 7 and 8). These results were consistent with the previous reports that NS1 is indispensable for the replication of HBoV1^9, 11^. However, transfection of pHBoV1NP1KO to HEK293T cells did not affect the replication of the viral genome (Fig. 1B, lane 6), which is different from a previous report^9^ that NP1 is essential for the replication of the virus.

### Point mutations in the NS1 ORF affected NP1 function

**Figure 2:**
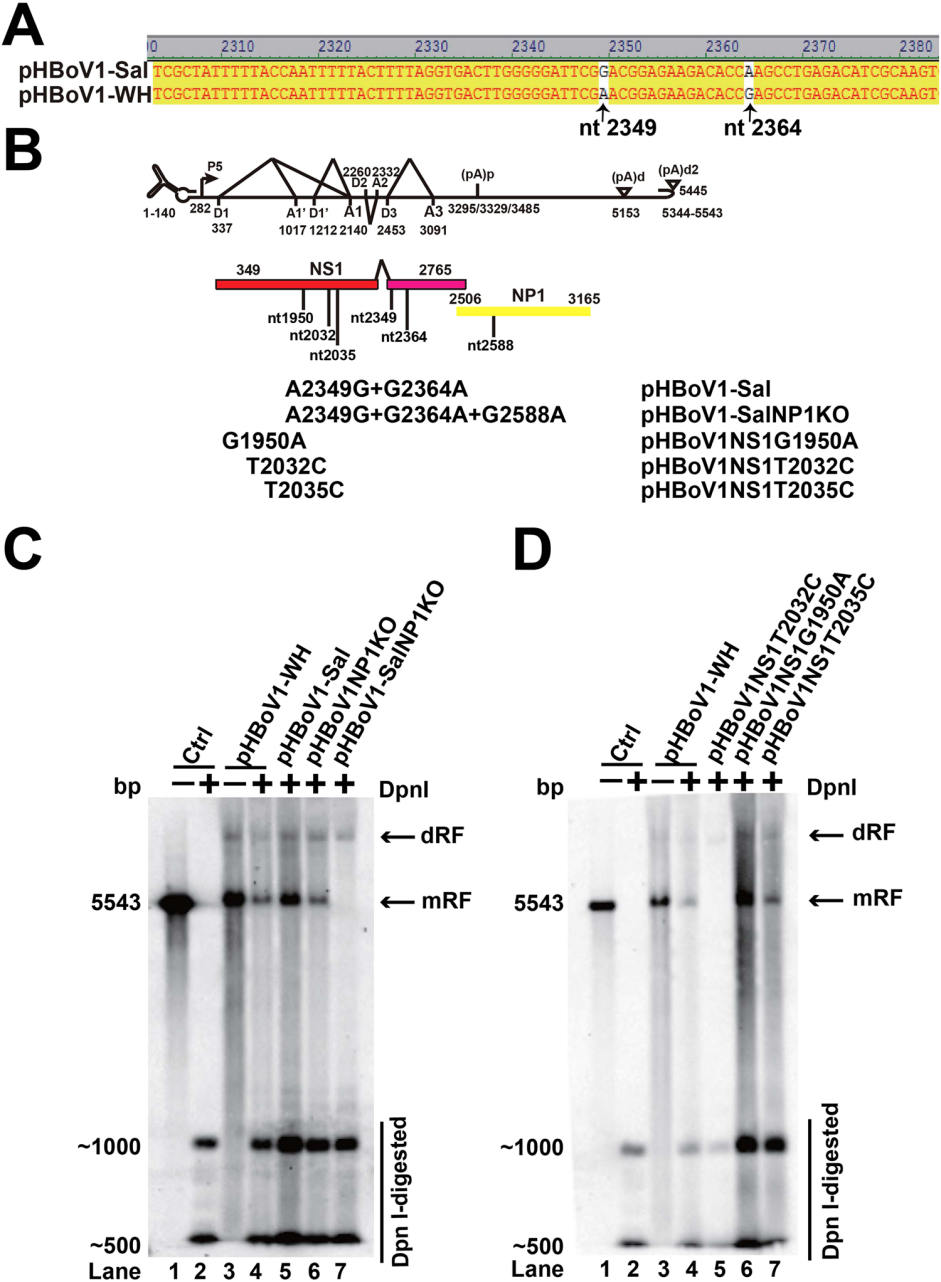
Mutations in the C-terminus of NS1 ORF influenced HBoV1 viral DNA replication and NP1 function. (**A**) Sequence alignment. The NS1 sequences of Wuhan isolate and Salvador strain were aligned. The mutations at nt 2349 and nt 2364 were indicated. (**B**) Diagram of mutated plasmids of NP1 and NS1. Mutations at nt 2349 and nt2364 in the Wuhan isolate were mutated to the sequence of Salvador strain. Random mutations in NS1 at nt 1950, 2035 and 2032 were indicated. (**C**,**D**) Southern blot analysis. Plasmids in panel B were transfected into HEK293T cells and the viral DNA was harvested 48 h after transfection. Dpn I digestion was performed and followed by Southern blot analysis. The signal was detected by ChemiDoc^TM^ MP imaging system.

To identify why NP1 knockouts did not affect viral DNA replication in pHBoV1-WH, we performed a sequence analysis between the Wuhan isolate and the reported infectious clone base from the Salvador1 isolate^9^, whose sequence was used as a reference in the following study. There were 3 nucleotide mutations (G2349A, A2364G, A4988G) that resulted in an amino acid mutation. The first two mutations (G2349A, A2364G) were located at the C-terminus of the NS1 coding region (Fig. 2A) and the third one was in the open reading frame of VP1/VP2 VP3. As VP1/VP2/VP3 knockout did not affect viral DNA replication, we focused on the two mutations G2349A and A2364G in the NS1 ORF. pHBoV1-Sal was constructed by mutating A2349G and G2364A based on pHBoV1-WH (Fig. 2B). The transfection of pHBoV1-Sal to HEK293T cells did not result in any change in the viral genomic replication (Fig. 2C, lane 5). However, the transfection of NP1KO plasmid pHBoV1-SalNP1KO to HEK293T cells resulted in poor viral DNA replication (Fig. 2C, lane 7), which was consistent with the previous report^9^, indicating that the two nucleotide mutations of NS1 were critical for the function of NP1 in genomic DNA replication.

NS1 is critical for HBoV genome replication. To obtain a single nucleotide mutation that abolishes the replication function of NS1, we made several mutations in the NS1 helicase domain. The mutation at nt 2032 from T to C in NS1 ORF resulted in the loss of Dpn I resistant band (Fig. 2D, lane 5), indicating that this single nucleotide is critical for the function of NS1. Two other random mutations, G1950A and T2035C, in the helicase domain were introduced into pHBoV1-WH. However, the viral replication was not affected by the mutations G1950A and T2035C (Fig. 2D, lanes 6 and 7). These results suggested that nt 2032 may be the key enzyme site of NS1^23^.

### The C-terminus mutations of NS1 affected NP1 function for viral DNA replication

The mutations G2349A and A2364G in the C-terminus of NS1 affected the function of NP1. To further check whether the mutations in the NS1 ORF affected the function of NP1, NS1 ORF was amplified from clinical nasopharyngeal aspirates (NPAs), and 13 out of 362 clinical samples were found to be positive. Sequence analysis showed 11 mutations among which 6 nonsense and 5 sense mutations were found in NS1 ORF (Table 1). We also analyzed 47 different strains from the previous reports^31^. Surprisingly, most of the mutations were located in the C-terminus of NS1 ORF; 7 sense mutations were discovered (Table 1).

**Table 1:** NS1 mutations from clinical samples or reported reference.

	nt	Salvador strain	Samples	Amino acid	Strains
NS1 mutations	2221	G	A	Asp----Asn	CQ1591 (ref. 28)
	2349	G	A	Asp----Asn	WH strain Swab56, 111, 149
	2358	G	A	Asp----Asn	Swab7 CQ586, AB48172, AB48174 (ref. 28)
	2364	A	G	Lys---Glu	WH strain Swab5, 7, 8, 9, 13, 14, 19, 20, 21, 23, 56, 149
	2418	G	A	Asp----Asn	CQ743, CQ1741, CQ1591, CQ474 (ref. 28)
	2421	C	T	Asp----Asn	CQ810, AB480175 (ref. 28)
	2430	C	T	Asp----Asn	CQ1741 (ref. 28)
	2474	T	A	Leu---Phe	Swab23, 111
	2574	G	A	Asp----Asn	CQ1741 (ref. 28)
	2676	A	G	Asn----Asp	Swab20, CQ1315 (ref. 28)

**Figure 3:**
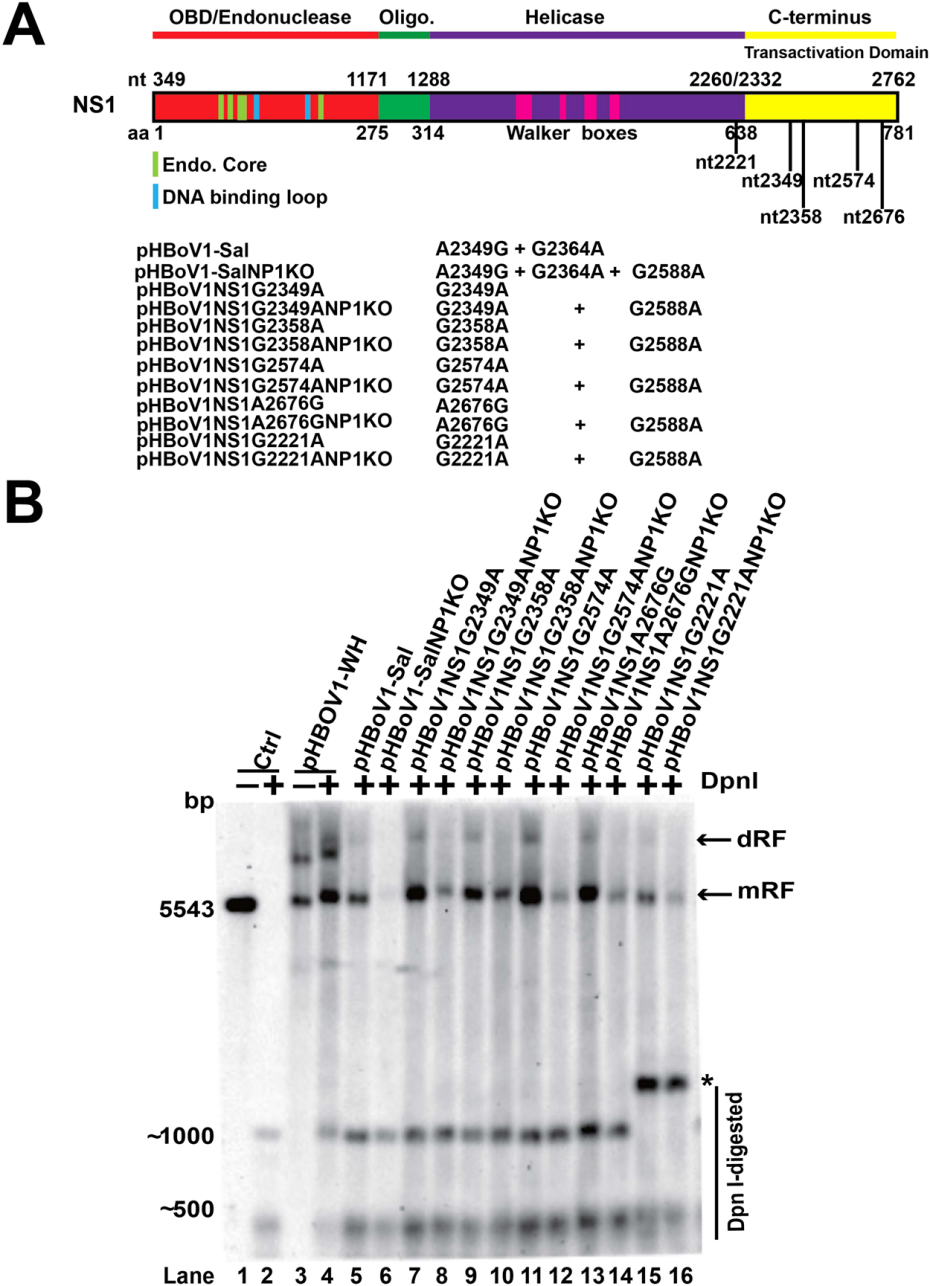
Mutations in the C-terminus of NS1 ORF affected NP1 function. (**A**) Diagram of NS1 ORF and NS1 mutated plasmids. NS1 ORF is presented with different functional domains, highlighting the nucleotide and amino acid position with numbers. Plasmids with mutations in the NS1 region from clinical samples or reports with or without NP1 knockout are shown. (**B**) Southern blot analysis. Plasmids in panel A were transfected into HEK293T cells. Low-molecular-weight DNA samples were harvested 48 h post-transfection. Southern blots were performed after Dpn I digestion and the signal was detected by ChemiDoc^TM^ MP imaging system. The size of Dpn I digested bands in lanes 15 and 16 is bigger because the Dpn I enzyme sites changed.

To further check the effect of the C-terminal sequence mutations on the viral DNA replication, the mutations, which caused an amino acid change in the NS1 region, were introduced into pHBoV1-Sal (Fig. 3A). The transfection of the NS1 mutated plasmids (pHBoV1NS1G2349A, pHBoV1NS1G2358A, pHBoV1NS1G2574A, pHBoV1NS1A2676G and pHBoV1NS1G2221A) into HEK293T cells did not lead to obvious replication change compared with pHBoV1-WH and pHBoV1-Sal (Fig. 3B, lanes 4, 5, 7, 9, 11, 13 and 15). This result suggested that the overall viral genomic replication was not altered by the mutations in the NS1 region. However, knockout NP1 ORF on the NS1 mutated plasmids resulted in decreased viral genome replication (Fig. 3B, lanes 6, 8, 10, 12, 14 and 16), suggesting that NP1 was necessary for the replication of viral genome when NS1 ORF contained certain mutations. Therefore, these results indicated that NS1 was a major player in viral DNA replication, and the mutations in the C-terminus of NS1 affected the function of NP1, which facilitated viral DNA replication.

### Mutations in the NP1 ORF had no effect on HBoV1 genomic DNA replication

**Figure 4:**
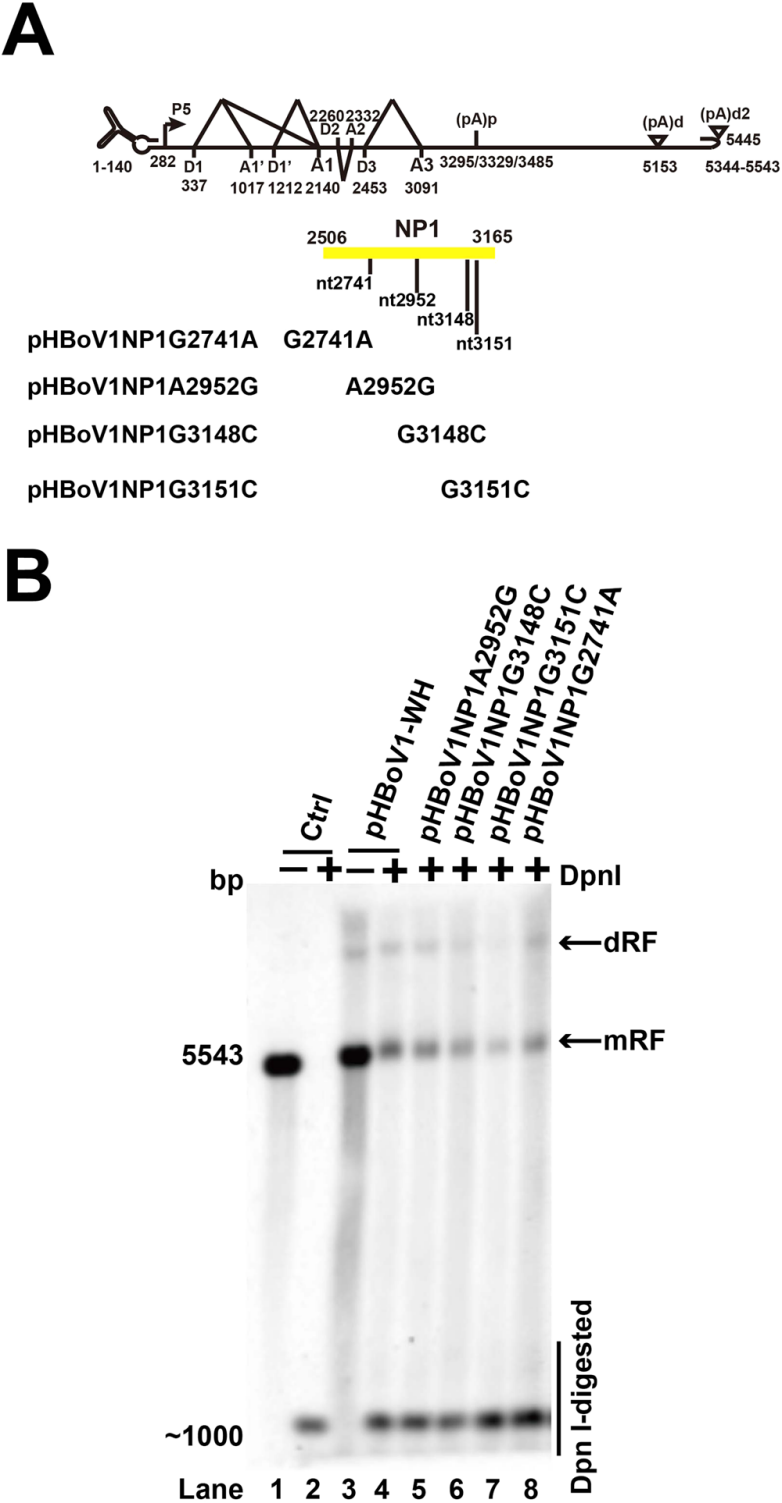
NP1 mutations did not affect HBoV1 genomic DNA replication. (**A**) NP1 ORF and mutations. Diagram of NP1 ORF and mutations from either clinical samples or reported cases are shown. (**B**) Southern blot analysis. Plasmids in panel A were transfected into HEK293T cells. DNA samples were harvested and digested with Dpn I. Southern blots were performed, and the signal was detected by ChemiDoc^TM^ MP imaging system.

Two NP1 mutations (G2741A and A2952G) caused lower viral load in patients^31^. G2741A mutation resulted in Ser to Asn amino acid mutation, while A2952G did not, indicating that the mutations in NP1 may affect viral replication. NP1 open reading frame was amplified from the 362 clinical samples, of which 13 were positive. Among the seven mutations detected, six were nonsense mutations, and 1 caused Ser to Asn amino acid mutation. To test the effect of NP1 point mutation on viral DNA replication, the reported NP1 mutations (G2741A and A2952G) and plasmids with two non-synonymous mutations (G3148C and G3151C) were constructed based on pHBoV1-WH and transfected into the HEK293T cells (Fig. 4A). Southern blot showed that the Dpn I resistant bands were not changed compared with pHBoV1-WH (Fig. 4B). The result implied that the detected or reported mutations in NP1 had no effect on the replication of HBoV1 genomic DNA.

### NP1 affected progeny virus production

**Figure 5:**
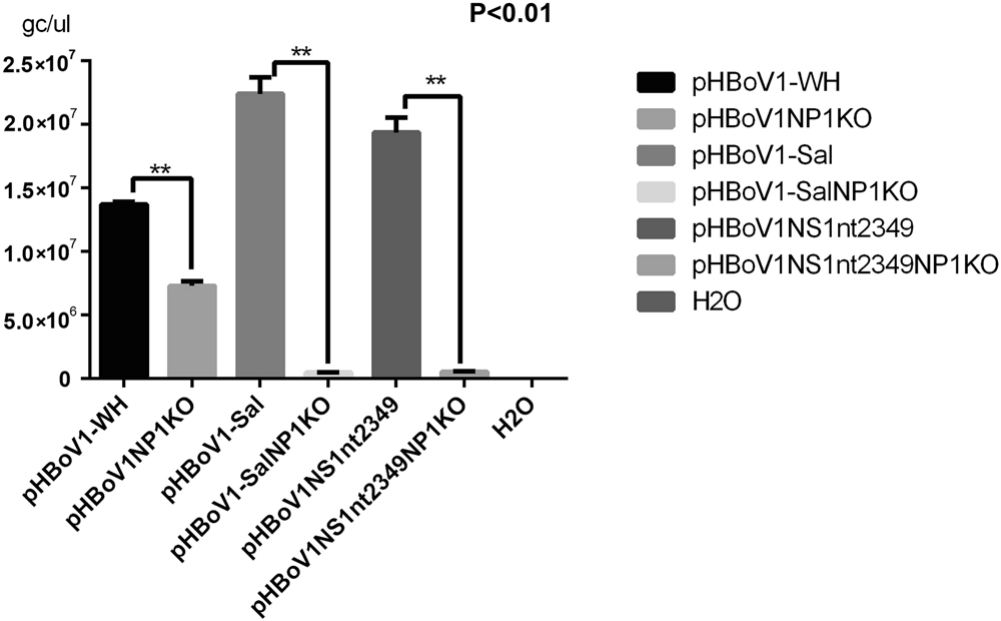
NP1 affected the progeny virus production. Quantitative PCR. Plasmids, as indicated, were transfected into HEK293 cells and progeny virus was harvested 48 h post-transfection. The genomic copy number of recombinant virus was quantified at least 3 times, and Student’s t-test was performed. P < 0.01 was significant and marked with**.

Mutations in the NP1 ORF did not affect the viral genome replication, and knockout NP1 in pHBoV1-WH did not influence the viral genome replication dramatically. It has been shown that NP1 plays a key role in the expression of capsid proteins^12^, which are critical for virus packaging, so we tested the effect of NP1 in progeny virus production. To this end, we transfected pHBoV1-WH, pHBoV1-Sal, pHBoVNS1nt2349 and their NP1 knockout mutants into HEK293T cells. The recombinant virus was harvested and quantified by PCR with NS1 primers. Interestingly, the genomic copy of the progeny virus was significantly decreased when NP1 was knocked out (Fig. 5), suggesting that NP1 may be involved in the virus particle packaging.

### UP1 was involved in the HBoV1 DNA replication

**Figure 6:**
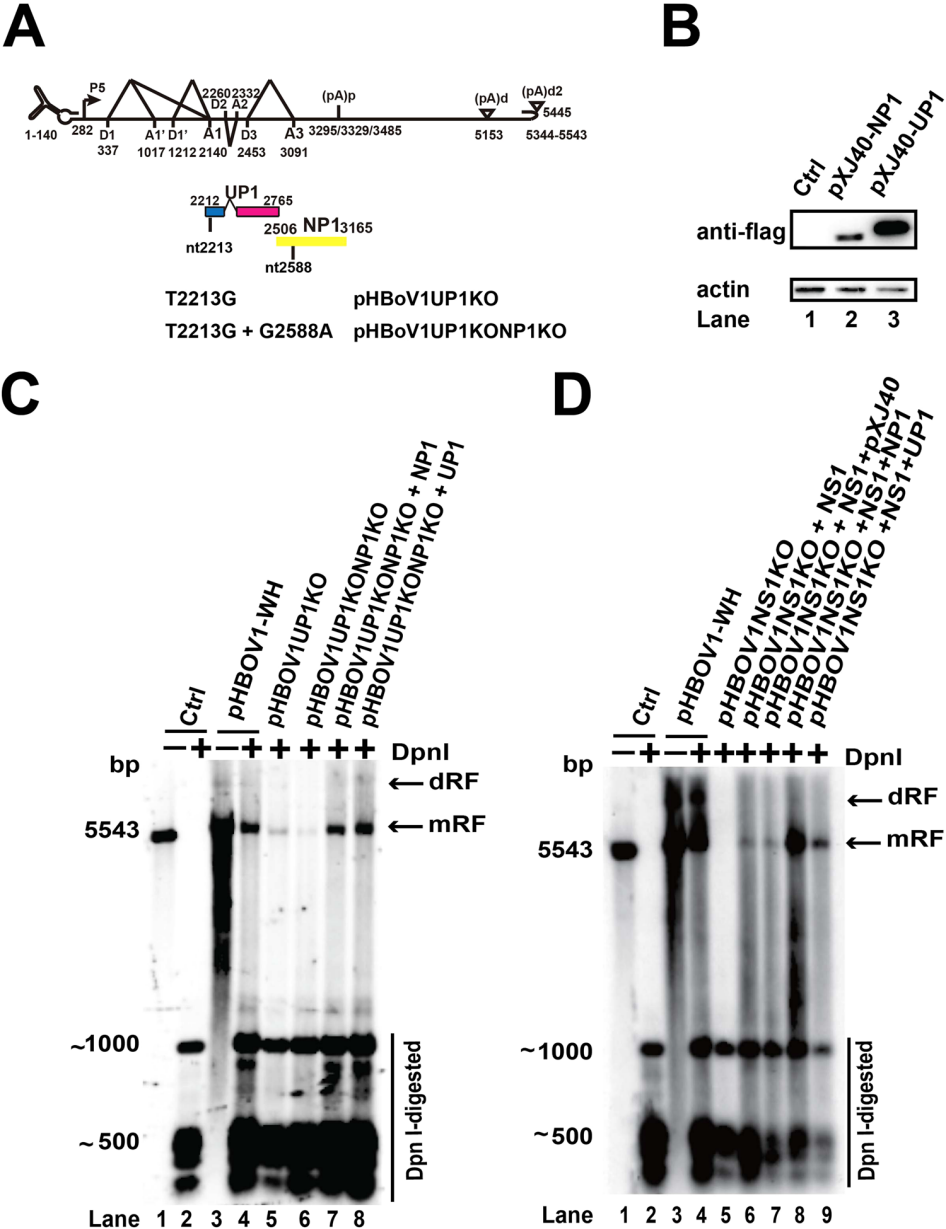
UP1 facilitated HBoV1 viral DNA replication. (**A**) Diagram of UP1 ORF and mutated plasmids. UP1 ORF was disrupted by a single nucleotide mutation. Both UP1 and NP1 ORFs were mutated as indicated. (**B**) Western blot. HEK293T cells were transfected with flag-tagged NP1 and UP1 expression plasmids. The transfected cells were lysed for Western blot analysis with flag and β-actin antibody. (**C** and **D**) Southern blot analysis. Plasmids in panel A were transfected into HEK293T cells together with UP1 or NP1 expressing plasmids. Hirt DNA was extracted, and Southern blot analysis was performed. The signal was detected by ChemiDoc^TM^ MP imaging system.

A non-structural protein UP1 was predicted from the transcription map of HBoV1 when AUG at nt 2212 was used^15^; however, its function is still unclear. The UP1 ORF is overlapped with the C-terminal sequence of NS1, and the first 17 amino acids of UP1 are identical to the amino acids 622 to 638 of NS1. All the point mutations that affected the function of NP1 overlapped with the open reading frame of UP1. We hypothesized that UP1 was involved in viral DNA replication. To test this hypothesis, UP1 knockout plasmid pHBoV1UP1KO was generated and transfected into HEK293T cells (Fig. 6A). Southern blot analysis showed that the Dpn I resistant band was not changed (Fig. 6C, lane 5), indicating that UP1 is not necessary for the replication of viral DNA. Transfecting the NP1 and UP1 double knockout plasmid pHBoV1UP1KONP1KO into HEK293T cells resulted in decreased replication of the viral genome (Fig. 6C, compare lane 6 to lane 5). However, co-transfection of NP1 or UP1 expression plasmid with pHBoV1UP1KONP1KO resulted in increased Dpn I resistant bands (Fig. 6C, lanes 7 and 8), indicating that both NP1 and UP1 promoted viral replication. To further confirm our result, we cotransfected NP1 or UP1 with pHBoVNS1KO and pXJ40-NS1 into HEK293T cells. The replication efficiency was quite low when pHBoVNS1KO and pXJ40-NS1 were transfected. However, transfection of UP1 or NP1 enhanced the replication efficiency (Fig. 6D, compare lanes 8 and 9 to lane 6), which confirmed that both NP1 and UP1 facilitated the genomic DNA replication of HBoV1.

### Identification of *cis-*elements that regulate HBoV1 viral DNA replication

**Figure 7:**
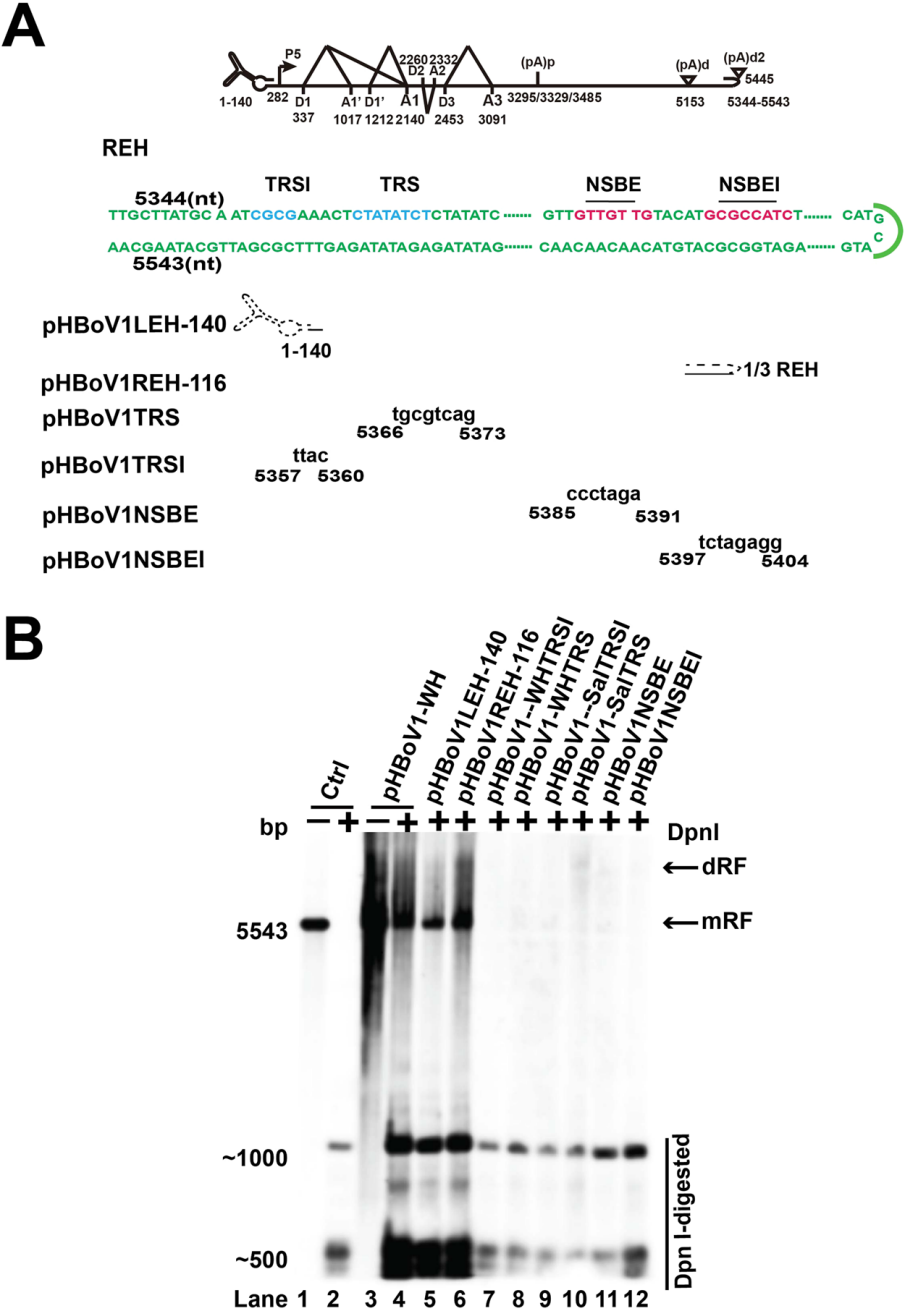
Identification of the *cis-*elements that regulate HBoV1 viral DNA replication. Diagram of LEH, REH, *trs* and NS1 binding elements mutated plasmids. LEH: Left end hairpin. REH: right end hairpin. *trs*: terminal resolution site. (**B**) Southern blot analysis. Hirt DNA was harvested from HEK293T cells transfected with plasmids in panel A. Southern blot analysis was performed as described in Fig. 1B.

LEH of HBoV1 is not required for the replication of the viral genome. The REH and 3′ NCR play an important role in viral genome replication^26^. Deleting the entire LEH or 2/3 REH from HBoV1-WH did not change the viral genome replication (Fig. 7B, lanes 5 and 6), which is consistent with a previous report^26^. The nicking site was reported as CTATATCT, and mutating this motif on pHBoV1-WH or pHBoV1-Sal inhibited the replication of viral DNA (Fig. 7B, lanes 8 and 10). In addition to this motif, we found that mutations in nt 5357 to nt 5360 (CGCG) also inhibited the replication of viral DNA (Fig. 7B, lanes 7 and 9), indicating that this motif also played a role in viral DNA replication. Mutation of NS1 binding elements (NSBE), which consist of four repeats of TGT, resulted in the decreased replication of viral genome (Fig. 7B, lane 11). However, mutations in nt 5397 to nt 5404 (GCGCCATC) also resulted in decreased replication of viral genome (Fig. 7B, lane 12). These results suggested that in addition to the reported TRS and NSBE, the CGCG and GCGCCATC motifs also play an important role in viral DNA replication.

## Discussion

In the present study, we found that mutations in clinical NPA samples in the C-terminus of NS1 ORF did not affect the overall viral genome replication. However, knocking out NP1 in the NS1 mutated clones resulted in decreased replication efficiency. These results suggested that the C-terminus played a role in viral replication, and NP1 facilitated the replication of the viral genome. Further study showed that UP1, whose ORF overlaps with the C-terminus of NS1, also promoted viral genome replication. Finally, we identified potential *trs* and NS1 binding elements in addition to the reported *cis* elements^26^.

Non-structural protein NS1 of parvoviruses is multifunctional and essential for viral replication. Moreover, knocking out NS1 resulted in a replication defect in the viral genome. In the present study, knocking out NS1 by point mutation resulted in the loss of HBoV1 genome replication, which is consistent with a previous report^9^. Mutations in the C-terminus were found in the Wuhan isolate, and Salvador1 isolate and in the clinical samples from the swabs of children aged 2 to 5 years. These mutations did not affect viral genome replication. The results suggested that mutations in the NS1 C-terminus did not affect the replication efficiency. To further prove whether this region is important for the replication of the viral genome, we transfected pHBoV1NS1-70, which did not contain the NS1 C-terminus of pHBoV1-WH. No viral genome replication was detected (data not shown). The results suggested that C-terminus was important for viral replication.

Parvovirus infection induces DNA damage response, which facilitates the replication of the viral genome^32^. HBoV1 can be cultured in CuFi-8 and human airway epithelial (HAE) or Calu-3 cells. The infection of non-dividing human airway epithelial (HAE) cells by HBoV1 induces a DNA damage response in which ATM, ATR and DNA-PKcs are activated^33^. The DNA repair polymerases are involved in viral genome amplification^33^. The Calu-3 cells start dividing, but it is unclear whether DDR is induced upon HBoV infection. The transactivation domain of NS1 can induce the G2/M arrest and apoptosis during parvovirus B19 virus infection^34^. The NS1 mutations in HBoV in our study were located in the transactivation domain, suggesting that NS1 may be involved in the DNA damage response, which warrants further investigation.

The unique feature of *bocaparvovirus* is the expression of nuclear protein NP1, which is involved in viral DNA replication^9, 19^, RNA polyadenylation^9, 12, 21^ and viral capsid protein expression^9, 12^. NP1 of MVC has been reported to participate in RNA splicing^11, 21^. Knockout NP1 resulted in viral genome reduction^9, 19^. However, a NP1 knockout in pHBoV1-WH did not affect the replication efficiency. Mutations in the two nucleotides at the C-terminus of NS1 on pHBoV1-WH resulted in no change in the overall viral replication. However, knockout NP1 resulted in the loss of replication. These results suggested that the two mutations in the NS1 region affected the function of NP1 and revealed that NS1 and NP1 orchestrated the regulation of viral genome replication.

To further test the function of NP1 on viral genome replication, NP1 sequences were amplified from clinical samples. Only one previously reported mutation^31^, which caused an amino acid change, was detected in 362 clinical samples. The mutation did not affect the replication efficiency of the viral genome. We also tested other reported NP1 mutants, and those mutants did not affect the replication of the viral genome, indicating that NP1 mutation from the clinical samples did not affect the replication efficiency.

NP1 regulates the expression of capsid proteins^12^, which are critical for virus packaging. To test the effect of NP1 on progeny virus production, we made a recombinant virus with or without NP1 knockout and then quantified the copy number of the progeny viral DNA by quantitative PCR. Interestingly, knocking out NP1 resulted in decreased progeny virus production, suggesting that NP1 plays a role in viral packaging. However, the detailed mechanism is unknown and warrants further study.

The UP1 ORF is located in the C-terminus of NS1. Knocking out the UP1 ORF without NP1 knockout did not affect viral genome replication. Transfection of the NS1 expression plasmids with pHBoV1NS1KO resulted in low viral genome replication efficiency. Co-transfection with UP1 increased the efficiency, indicating that UP1 promoted the viral genome replication.

The terminal repeats of parvovirus are important for viral genome replication. Deletion of entire LEH or 2/3 of REH did not change the replication efficiency of the viral genome, which is consistent with a previous report^26^ that LEH is not required for viral genome replication, and the minimal requirement for HBoV1 DNA replication contains a 46-nt replication origin. The replication origin of HBoV1 contains a nicking site [5′-CT(A/T)ATCT-3′] and an NS1 binding site, which is composed of four TGT repeats^26^. However, NS1 did not bind to this replication origin *in vitro*. We identified a motif GCGT located at the 5′ end of the right end hairpin and a GC-rich element required for viral genome replication, which could be a part of the *cis*-element.

Parvovirus genomic DNA replication is important for its life cycle. DNA replication, which overcomes the blockage of full-length mRNA production by the polyadenylation site at the proximal site, is a limiting step for B19V infection^35^. NS1 plays a major role in viral genome replication. Our results showed that NP1 and UP1 facilitated viral DNA replication. We also found that point mutations in the C-terminus of NS1 affected the function of NP1, although the detailed mechanism is still unclear. The importance of the C-terminus of NS1 in the replication of viral DNA provided new insights into blocking viral infection in the future.

## Materials and Methods

### Cell line and cell culture

HEK293T (ATCC, CRL-11268) cells were cultured in Dulbecco’s modified Eagle’s medium (DMEM, GIBCO) containing 10% FBS (GIBCO) at 37 °C with 5% CO_2_.

### Clinical sample preparation

The clinical HBoV1 swabs were provided by Union Hospital and Guangzhou Women and Children’s Medical Center. All samples and patient medical history were collected with informed consent from the consent of guardians. The study protocol was approved and carried out in accordance with the guidelines and regulations of Wuhan Institute of Virology, Chinese Academy of Sciences. Demographic and epidemiological data of patients were kept anonymous and not included in the study database. Viral genomic DNA was extracted with QIAamp DNA Mini Kit (QIAGEN, 51304) according to the manufacturer’s protocol.

### NS1 and NP1 amplification

NS1 ORF was amplified from the clinical viral genomic DNA by using primers (Forward: 5′-CCCATCGATTGGCCAAGGCAATCGTCCAAG-3′, Reverse: 5′-CGCGTCGACCTATTGTCTTTTTTCCCCGATGTAC-3′). NP1 ORF was amplified with primers (Forward: 5′-CCGCTCGAGATGAGCTCAGGGAATATGAAAGAC-3′, Reverse: 5′-GGAAGATCTTTAATTGGAGGCATCTGCTTC-3′). The PCR amplifications were carried out with Easytaq DNA Polymerase.

### Transfection

HEK293T cells grown in 60-mm dishes were transfected with 2 µg of HBoV1 DNA fragments or plasmids with Lipofectamine 2000 reagents (Invitrogen, 11668-019) according to the manufacturer’s instructions.

### Plasmid construction

(i) pHBoV1-WH and mutants: pHBoV1-WH, pHBoV1NS1KO, pHBoV1NP1KO, pHBoV1VP1KO and pHBoV1VP2/3KO have been described previously^9^.
  pHBoV1UP1KO was constructed by mutating nt 2213 from T to A, which resulted in early translation termination of the open reading frame. pHBoV1UP1KONP1KO was generated by a mutation in nt 2588 from G to A based on pHBoV1UP1KO. pHBoV1NS1-70 was made by mutating nt 2347 from C to A. pHBoV1NS1G1950A, pHBoV1NS1T2032C, pHBoV1NS1T2035C, pHBoV1NP1G2741A, pHBoV1NP1A2952G, pHBoV1NP1G3148C and pHBoV1NP1G3151C were constructed by mutating nt 1950 from G to A, nt 2032 and nt 2035 from T to C, nt 2741 from G to A, nt 2952 from A to G, nt 3148 and nt 3151 from G to C, respectively.
(ii) pHBoV1-Sal and mutants: pHBoV1-Sal was constructed by a mutation in nt 2349 from A to G and nt 2364 from G to A based on pHBoV1-WH. pHBoV1NS1G2349A, pHBoV1NS1G2358A, pHBoV1NS1G2574A, pHBoV1NS1G2221A and pHBoV1NS1A2676G were generated by mutating nt 2349, nt 2358, nt 2574 and nt 2221 from G to A, nt 2676 from A to G, respectively.
(iii) Inverted terminal repeat (ITR) mutants: pHBoV1LEH-140 has been described^36^. pHBoV1REH-116 was constructed by truncating the last 116 nt of the HBoV1 genome based on pHBoV1LEH-140, which does not contain the LEH.
(iv) Construction of plasmids to test NS1 binding elements and terminal resolution site: pHBoV1-NSBE and pHBoV1-NSBEI were constructed by mutating nt 5385 to nt 5391 from GTTGTTG to CCCTAGA, nt 5397 to nt 5404 from GCGCCATC to TCTAGAGG, respectively. pHBoV1TRS and pHBoV1TRSI were constructed by mutating nt 5366 to nt 5373 from CTATATCT to TGCGTCAG and nt 5357 to nt 5360 from CGCG to TTAC respectively.
(v) Construction of NS1, NP1, UP1 expression plasmids: The open reading frames of NS1, NP1 and UP1 were inserted into pXJ40-Flag vector.

### Isolation of low-molecular-weight (Hirt) DNA and Southern blot analysis

DNA of low molecular weight (Hirt DNA) was isolated to detect the replication forms^18, 35^. After HBoV plasmids transfection, the cells were washed twice with phosphate-buffered saline and lysed in 2% sodium dodecyl sulfate followed by proteinase K (0.5 mg/ml) treatment. DNA was harvested and subjected to Dpn I digestion. Digested DNA fragments were separated by 1% agarose gel and transferred to Hybond N^+^ membrane. The blots were hybridized with the pHBoV1-WH genome probe spanning from nt 1 to nt 5543 with DIG High Prime DNA Labeling and Detection Starter Kit II (Roche) according to the manufacturer’s protocol. Signals were detected with ChemiDoc^TM^ MP imaging system (BIO-RAD).

### Recombinant HBoV virus production

HEK293T cells were cultured on six 100-mm plates and transfected with 6 µg recombinant plasmids per dish using the Lipofectamine 2000 reagents (Invitrogen). After being maintained for 48 h at 5% CO_2_ and 37 °C, the cells were collected, resuspended in 6 ml PBS, and lysed by subjecting to four rounds of freezing (−80 °C) and thawing (37 °C). Benzonase (Novagen) was added to the mixture (50 U/ml, final concentration) and incubated for 30 min at 37 °C. The cell lysate was then spun at 10,000 rpm for 30 min. The supernatant was loaded into a 13-ml centrifuge tube with a continuous CsCl gradient and spun in a SW41 rotor (Beckman) at 36,000 rpm for 36 h. The recombinant virus was collected and desalted by centrifugation through the 100 KDa filter (Millipore) according to the manufacturer’s instructions. Viral DNA was extracted with the TIANamp Virus DNA/RNA Kit (TIANGEN, Beijing, China) according to the manufacturer’s protocol.

### Quantitative PCR and statistical analysis

Quantitative PCR of recombinant HBoV genomic copy was performed with NS1 primers (NS1-F: 5′-TGCAGACAACGCCTAGTTGTTT-3′, NS1-R: 5′-CTGTCCCGCCCAAGATACA-3′) and SYBR Green real time master mix (Toyobo). The experiments were repeated at least 3 times. Student’s *t*-test was applied to determine the differences in the viral genome copy. p < 0.01 was significant and marked with**.

### Western blot

Cell lysates were prepared 48 h after transfection and separated by SDS-12%PAGE followed by transfer to nitrocellulose. Protein detection was carried out using standard protocols with anti-Flag (Sigma, F1804) and anti-actin (Santa Cruz Biotechnology, sc-47778) antibodies. Luminescent signals were detected with ChemiDoc^TM^ MP imaging system (BIO-RAD).

## Electronic supplementary material

Supplementary Information
