# Supplementary material for: Mutations in the C-terminus of HBoV NS1 affect the function of NP1
Source: Sci Rep. 2017 Aug 7;7:7407. doi: 10.1038/s41598-017-06513-4 (PMC5547040; doi:10.1038/s41598-017-06513-4)
Supplement: Supplementary file 1 — Supplementary Information [file 41598_2017_6513_MOESM1_ESM.pdf]

Mutations in the C-terminus of HBoV NS1 affect the function of NP1

Junmei Zhang[1,2], Yan Bai[3], Bing Zhu[4], Sujuan Hao[1,2], Zhen Chen[1], Hanzhong Wang[1], Wuxiang Guan[1 *]

1. Center for Emerging Infectious Diseases, Wuhan Institute of Virology, Chinese Academy of Sciences, Wuhan, Hubei, 430071, China

2. University of Chinese Academy of Sciences, Beijing, 100049, China

3. Pediatric department of Union Hospital, Tongji Medical College, Huazhong University of Science and Technology, Wuhan, China

4. Center Laboratory, Guangzhou Women and Children's Medical Center, Guangzhou, 510120, P. R. China.

# Address correspondence to WG (guanwx@wh.iov.cn or guanwx2000@hotmail.com)

Uncropped data for Figure 1

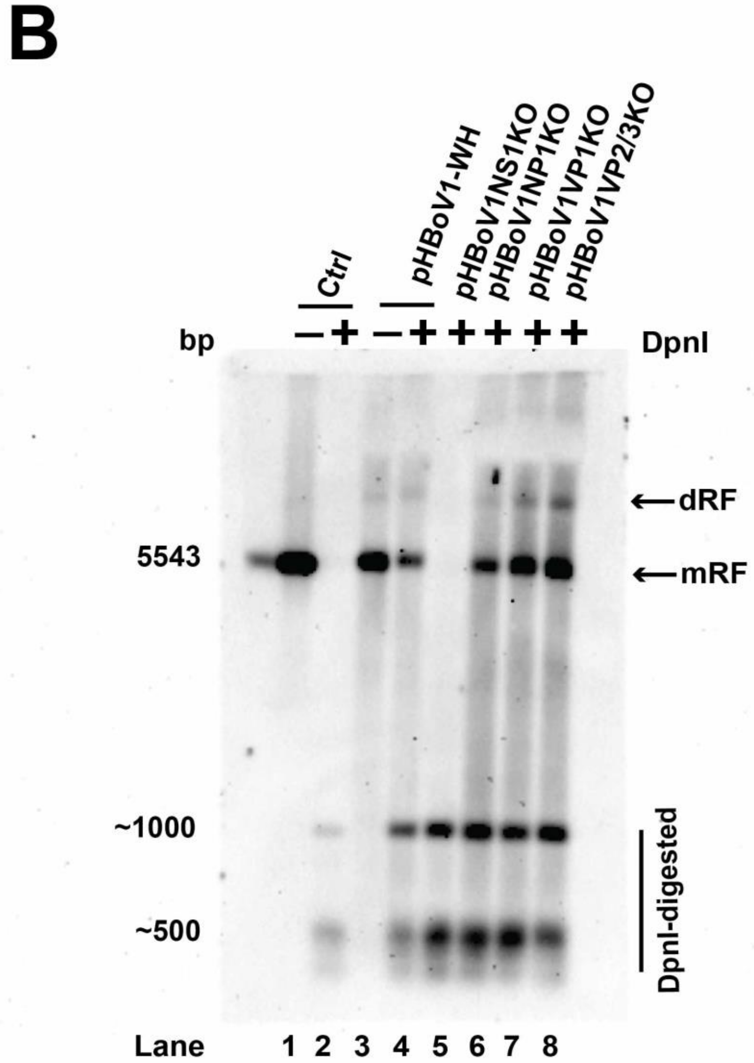

Figure 1

Uncropped data for Figure 2

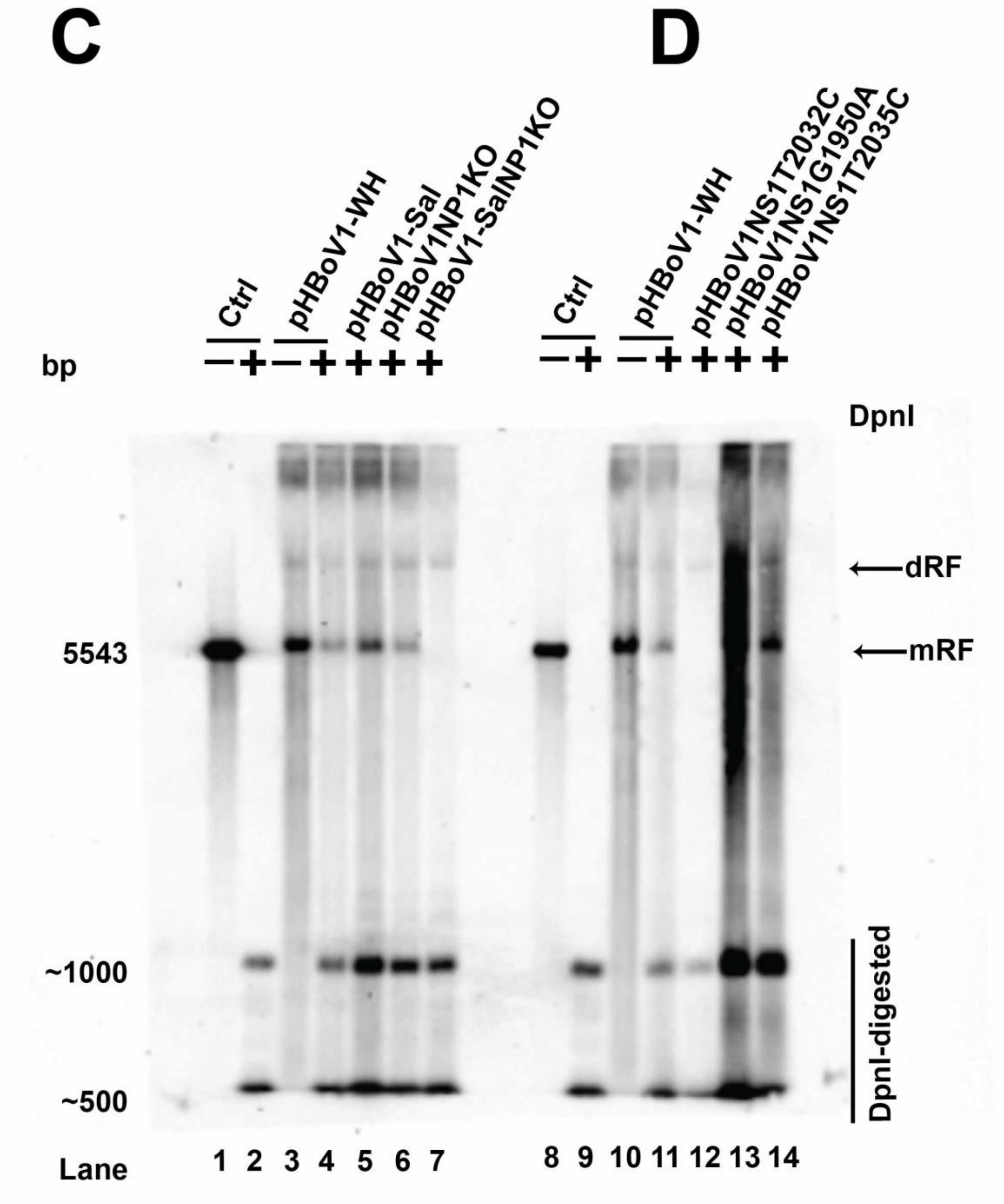

Figure 2

Uncropped data for Figure 3

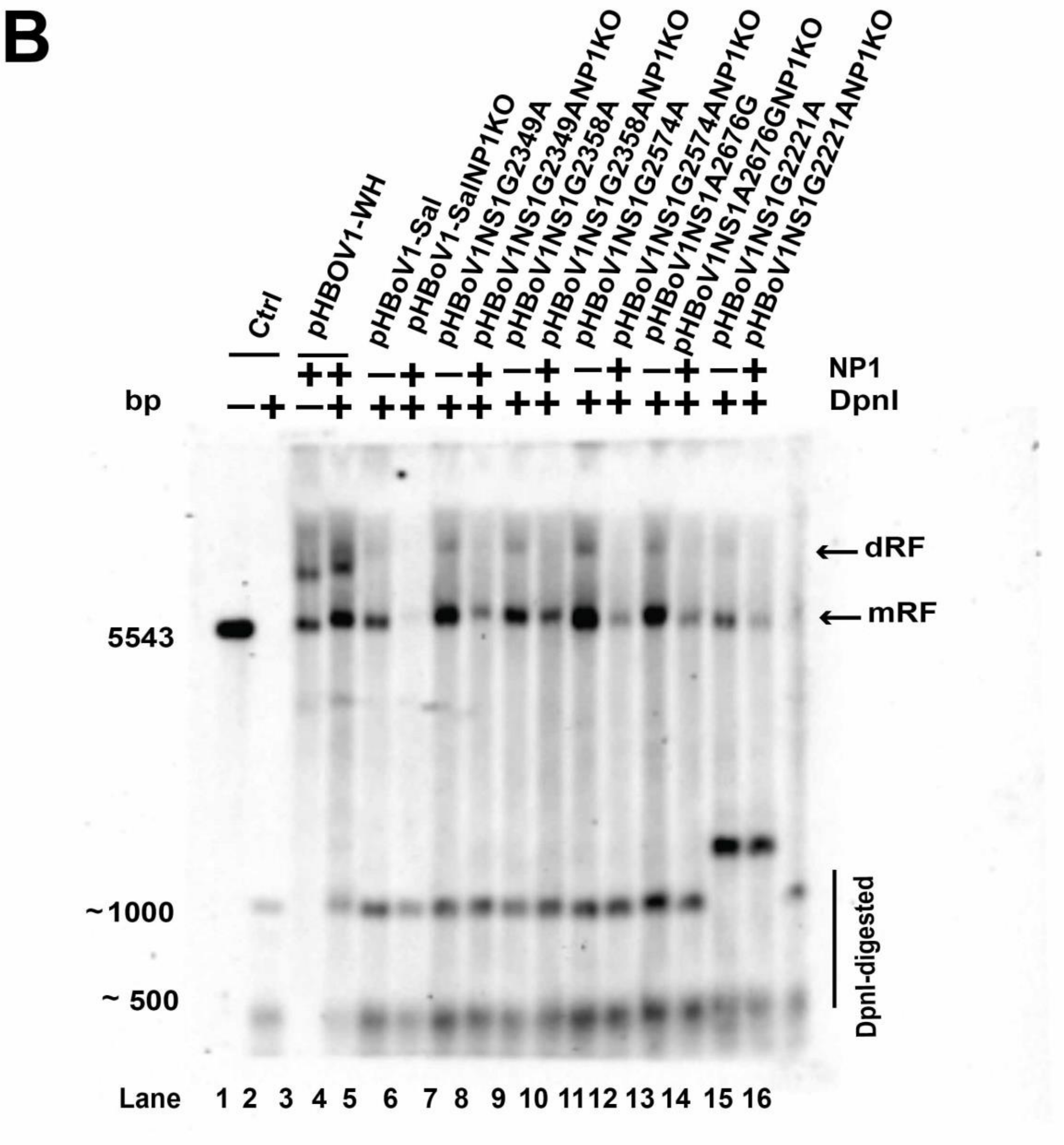

Figure 3

Uncropped data for Figure 4

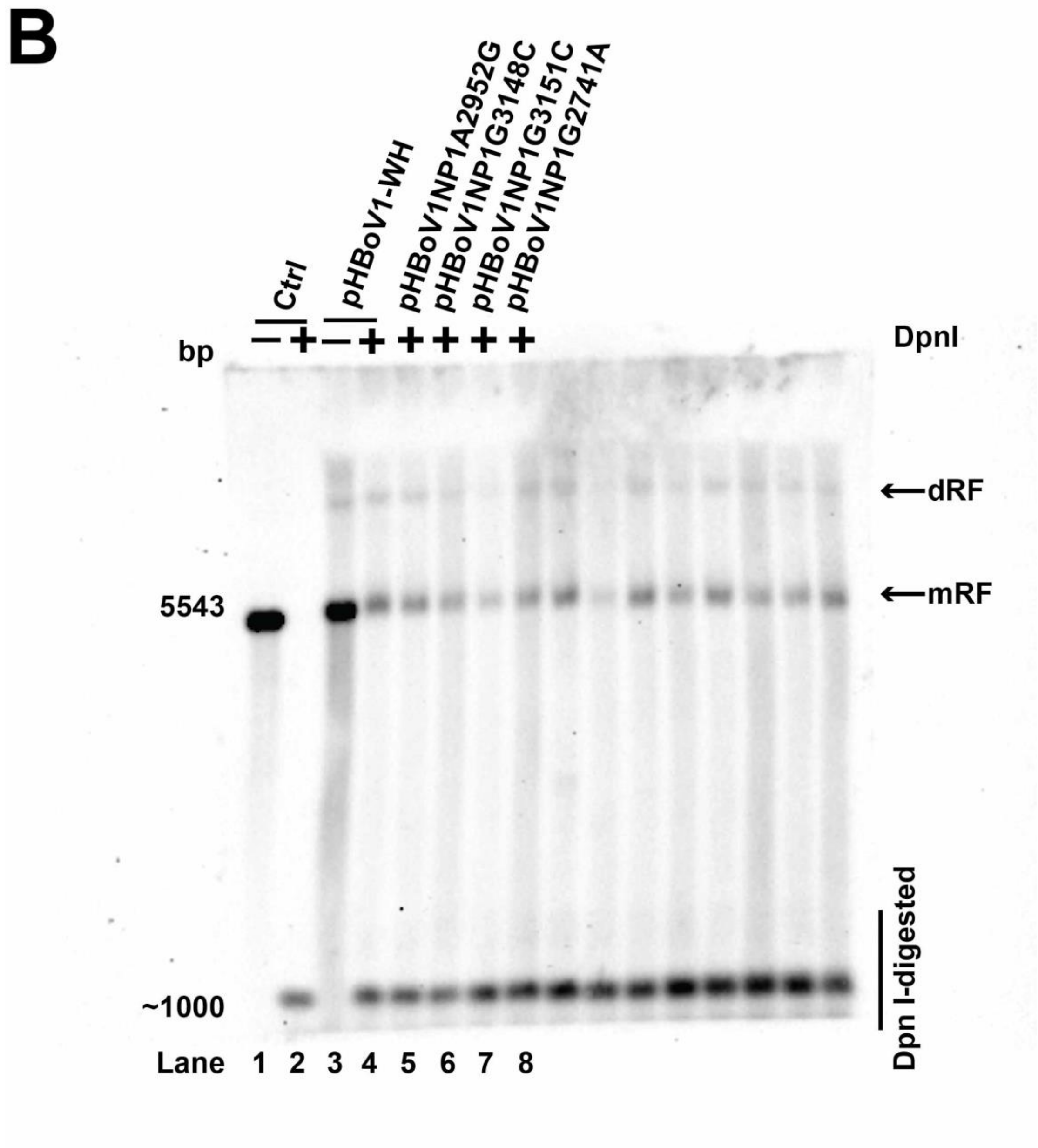

Figure 4

Uncropped data for Figure 6

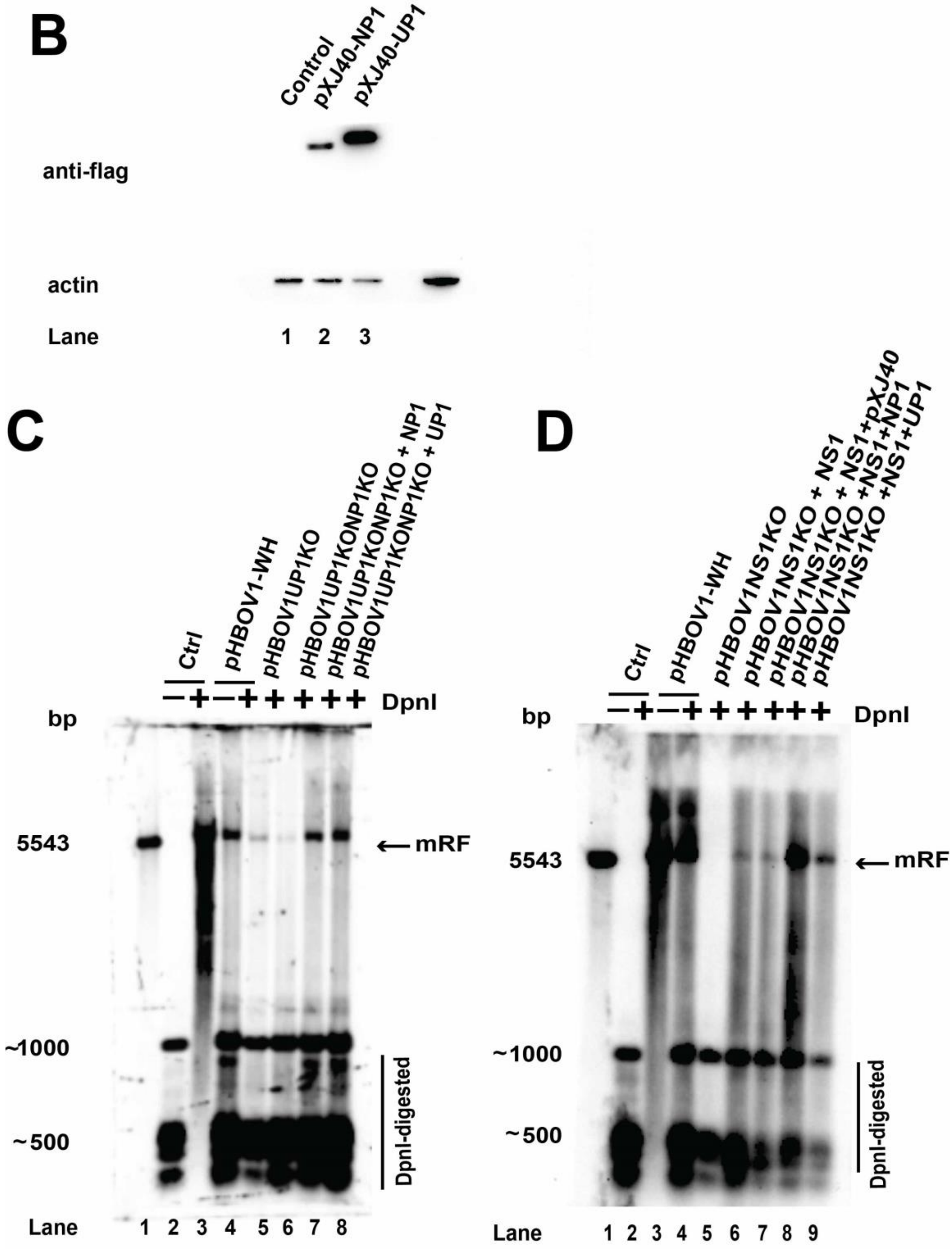

Figure 6

Uncropped data for Figure 7

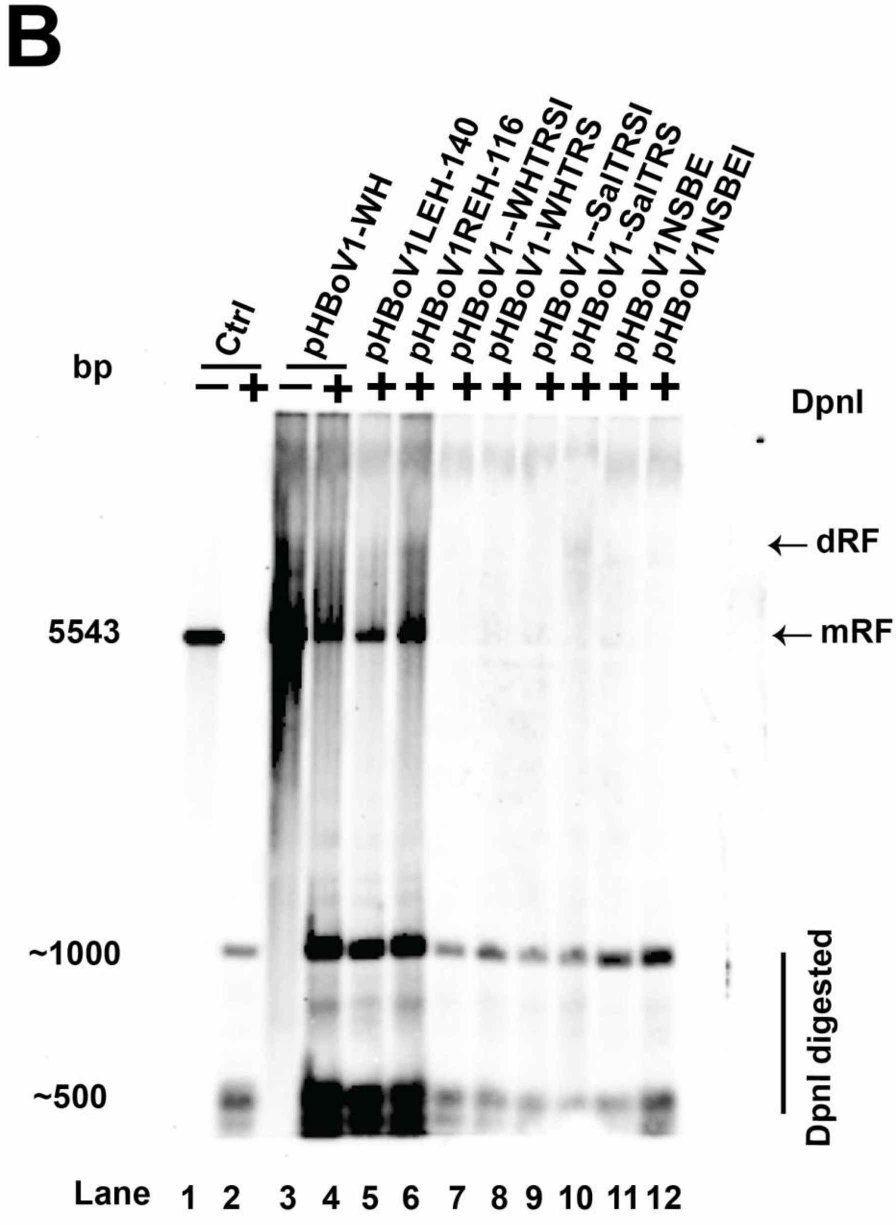

Figure 7
